# Multimorbidity incidence following hospitalization for SARS-CoV-1 infection or influenza over two decades: a territory-wide retrospective cohort study

**DOI:** 10.1038/s41533-025-00424-y

**Published:** 2025-03-25

**Authors:** Cuiling Wei, Chor Wing Sing, Eric Yuk Fai Wan, Ching Lung Cheung, Ian Chi Kei Wong, Francisco Tsz Tsun Lai

**Affiliations:** 1 Centre for Safe Medication Practice and Research, Department of Pharmacology and Pharmacy, Li Ka Shing Faculty of Medicine, The University of Hong Kong, Pok Fu Lam, Hong Kong SAR, China; 2 Laboratory of Data Discovery for Health (D24H), Hong Kong Science Park, Sha Tin, Hong Kong SAR, China; 3 Department of Family Medicine and Primary Care, School of Clinical Medicine, Li Ka Shing Faculty of Medicine, The University of Hong Kong, Pok Fu Lam, Hong Kong SAR, China; 4 Advanced Data Analytics for Medical Science (ADAMS) Limited, Hong Kong SAR, China; 5 Aston Pharmacy School, Aston University, Birmingham, England UK

**Keywords:** Epidemiology, Outcomes research

## Abstract

An infection of SARS-CoV-1, the causative agent of Severe Acute Respiratory Syndrome (SARS), may be followed by long-term clinical sequala. We hypothesized a greater 20-year multimorbidity incidence in people hospitalized for SARS-CoV-1 infection than those for influenza during similar periods. We conducted a retrospective cohort study using a territory-wide public healthcare database in Hong Kong. All patients aged ≥15 hospitalized for SARS in 2003 or influenza in 2002 or 2004 with no more than one of 30 listed chronic disease were included. Demographics, clinical history, and medication use were adjusted for in the inverse-probability-of-treatment-weighted Poisson regression analyses. We identified 1255 hospitalizations for SARS-CoV-1 infection and 687 hospitalizations for influenza. Overall crude multimorbidity incident rates were 1.5 per 100 person-years among SARS patients and 5.6 among influenza patients. Adjusted multimorbidity incidence rate ratio (IRR) was estimated at 0.78 [95% confidence interval (CI), 0.70–0.86) for SARS patients compared with influenza patients. Analysis by follow-up period shows a potentially greater risk among SARS patients in the first year of follow-up (IRR 1.33, 95% CI 0.97–1.84), with the risk in influenza patients increasing in subsequent years. Subgroup analyses by age and sex showed consistent results with the main analysis that SARS-CoV-1 infection was not followed by a higher incidence of multimorbidity than influenza. Notable differences in the patterns of multimorbidity were identified between the two arms. To conclude, we found no evidence of a higher multimorbidity incidence after hospitalization for SARS than for influenza over the long-term.

## Introduction

SARS-CoV-1, the causative agent of severe acute respiratory syndrome (SARS), is an ancestral viral strain of SARS-CoV-2, the causative agent of COVID-19^1^. Unlike SARS-CoV-2 however, it has not caused remotely as disastrous an impact on humanity as the COVID-19 pandemic did^2^. The only notable SARS epidemic took place in China in early 2003, before surprisingly dying down in the same year without a noticeable re-emergence^3^.

Like recent studies on long COVID syndrome and other adverse sequalae^4,5^, there have been reports concerning prognosis of SARS relative to other respiratory infections, apart from a very high case-fatality rate from SARS, i.e., acute lung injury and acute respiratory distress syndrome^6^. Indeed, survivors of SARS are reported to have an elevated risk of developing long-term conditions, such as dyspnea, pulmonary fibrosis, anxiety and depression^6^. It is, thus, probable that they are also at a heightened risk of developing multimorbidity, commonly referred to as the cooccurrence of two or more chronic conditions in an individual^7,8^. The incidence and prevalence of multimorbidity, which is consistently associated with poorer quality of life^9^, more healthcare utilization^10,11^, and greater mortality risks^12^, are highly indicative of the chronic health care burden in a health system^10,13^. Nevertheless, there is no existing study examining the long-term impact of SARS on the incidence of multimorbidity.

Hong Kong was one of the places affected most significantly by SARS in 2003, with more than 1700 people being infected and nearly 300 people dying from it^14^, out of a 7-million population. With a unified public healthcare system under the Hospital Authority (HA) and comprehensive digitalized longitudinal clinical records, we aimed to conduct a retrospective cohort study to compare SARS survivors with patients hospitalized for influenza, over an observation period of two decades up to 2022. Given the previous research on the severe adverse sequalae following SARS^15^, we hypothesize a greater multimorbidity incidence following SARS-CoV-1 infection-related hospitalization compared with an influenza hospitalization which is commonly seen and routinely managed.

## Methods

### Study design and data source

We conducted a retrospective cohort study using the clinical records of a territory-wide database covering patients attending public healthcare facilities, which were maintained in the Clinical Data Analysis and Reporting System (CDARS) of the HA. This database has been used for numerous excellent large-scale epidemiologic studies previously^16,17^. The HA is the sole provider of public inpatient services and a major provider of public outpatient services in Hong Kong, covering more than 80% of all healthcare service users in the city. The vast majority of patients with chronic diseases are regularly followed up in HA facilities. All disease diagnoses in this study were identified using the International Classification of Diseases, Ninth Revision, Clinical Modification (ICD-9-CM) codes within CDARS. Previous research has validated this coding system’s reliability, with positive predictive values exceeding 85% for various diseases^18,19^.

### Cohort selection

As children under the age of 15 are less representative of the population at risk of developing multimorbidity, our cohort was defined as all people aged 15 or older who were hospitalized for influenza for the first time in 2002 or 2004 (as the influenza arm) or SARS in 2003 (as the SARS arm). Also, since the age distribution of SARS patients, the exposed group of interest, did not significantly skew towards children under 15, we selected this group of influenza patients with a similar demographic profile to enable a fair and meaningful comparison with the SARS group.

Deaths, co-existing influenza and SARS, or the occurrence of multimorbidity (the outcome of interest, please see the next section for more details) on or before the index date were used as exclusion criteria. We defined the index date, i.e., start of observation, as the date of discharge from influenza or SARS hospitalization, and followed the patients until the occurrence of the outcome, i.e., multimorbidity, all-cause mortality, or the end of data availability. To ensure inclusion of serious cases requiring a certain length of hospital stay, we excluded records with the same admission and discharge dates as these records were considered invalid because they indicated transient non-critical admissions or even mild conditions, or potential system input errors. In case of multiple hospitalizations, the latest episode was used as the index hospitalization. The influenza arm of the cohort was identified using the ICD-9-CM code: 487, while the SARS arm was identified from a previously established database maintained by the Department of Health for the purpose of a more comprehensive follow-up of patients^20^.

### Outcome

Multimorbidity was adopted as the primary study outcome, while specific listed chronic conditions were analyzed as secondary outcomes. A widely used list of 30 chronic conditions was used for the definition of multimorbidity^21^, with the corresponding ICD-9-CM codes shown in S1 Table. The 30 conditions were chosen to encompass a wide variety of diseases requiring different types of care and specialist attention, including alcohol misuse, asthma, atrial fibrillation, chronic heart failure, chronic kidney disease, chronic pain, chronic pulmonary disease, chronic viral hepatitis B, cirrhosis, dementia, depression, diabetes, epilepsy, hypertension, hypothyroidism, inflammatory bowel disease, irritable bowel syndrome, lymphoma, metastatic cancer, multiple sclerosis, myocardial infarction, non-metastatic cancer (breast, cervical, colorectal, lung, and prostate), Parkinson’s disease, peptic ulcer disease, peripheral vascular disease, psoriasis, rheumatoid arthritis, schizophrenia, severe constipation, and stroke or transient ischemic attack (TIA). The diagnosis of the second listed chronic condition in the patient was operationalized as the occurrence of multimorbidity.

### Statistical analysis

Incidence rate ratios (IRR) with 95% confidence intervals (CI) were estimated using multivariable Poisson regression analysis. Inverse probability of treatment weighting (IPTW) was used to balance between the two arms in terms of the baseline characteristics including age, sex, chronic condition and medicine use (S2 Table) at baseline. We calculated the standard mean difference for continuous variables and the proportion difference for dichotomous variables before and after weighting to examine the balance between the arms. We further included variables with a standard mean (SMD) or proportion difference that were greater than 0.1 in the Poisson regression as covariates. Subgroup analyses were performed separately by sex and age group, i.e., <40 or ≥40 years. Covariates were reweighed in every subgroup analysis.

### Sensitivity analysis

We conducted a series of sensitivity analyses to test for the robustness of findings. First, we included influenza hospitalizations in 2003, coinciding with the SARS epidemic in Hong Kong, to consider the outbreak’s potential influence on influenza cases that year. Second, index date was redefined as one year after the discharge date to capture only the post-acute effects. Third, we adopted three diseases as the definition of the multimorbidity outcome. Fourth, we performed multivariable Poisson regression to adjust for covariates instead of using IPTW to weigh the sample based on the covariates. Fifth, we conducted a competing risk regression adjusting for all-cause mortality as a potential competing risk outcome. Sixth, we operationalized antivirals and antibiotics only during the current episode as covariates and repeated the main analysis. Seventh, given the seasonality of influenza, we further stratified the influenza group into peak and non-peak season admissions. According to the Hong Kong Centre for Health Protection, the annual peak influenza seasons in the region occur from January to April and July to August^22^. We replicated multivariable models with such further stratification to detect any impact of influenza seasonality on the findings. Lastly, we stratified the observation period into less than one year, 1–5 years, 6–10 years and beyond 10 years to observe difference across periods.

All analyses were carried out using SAS version 9.4 and R software version 4.0.5. A two-sided *p*-value < 0.05 was taken as significant in this study.

## Results

**Fig. 1:**
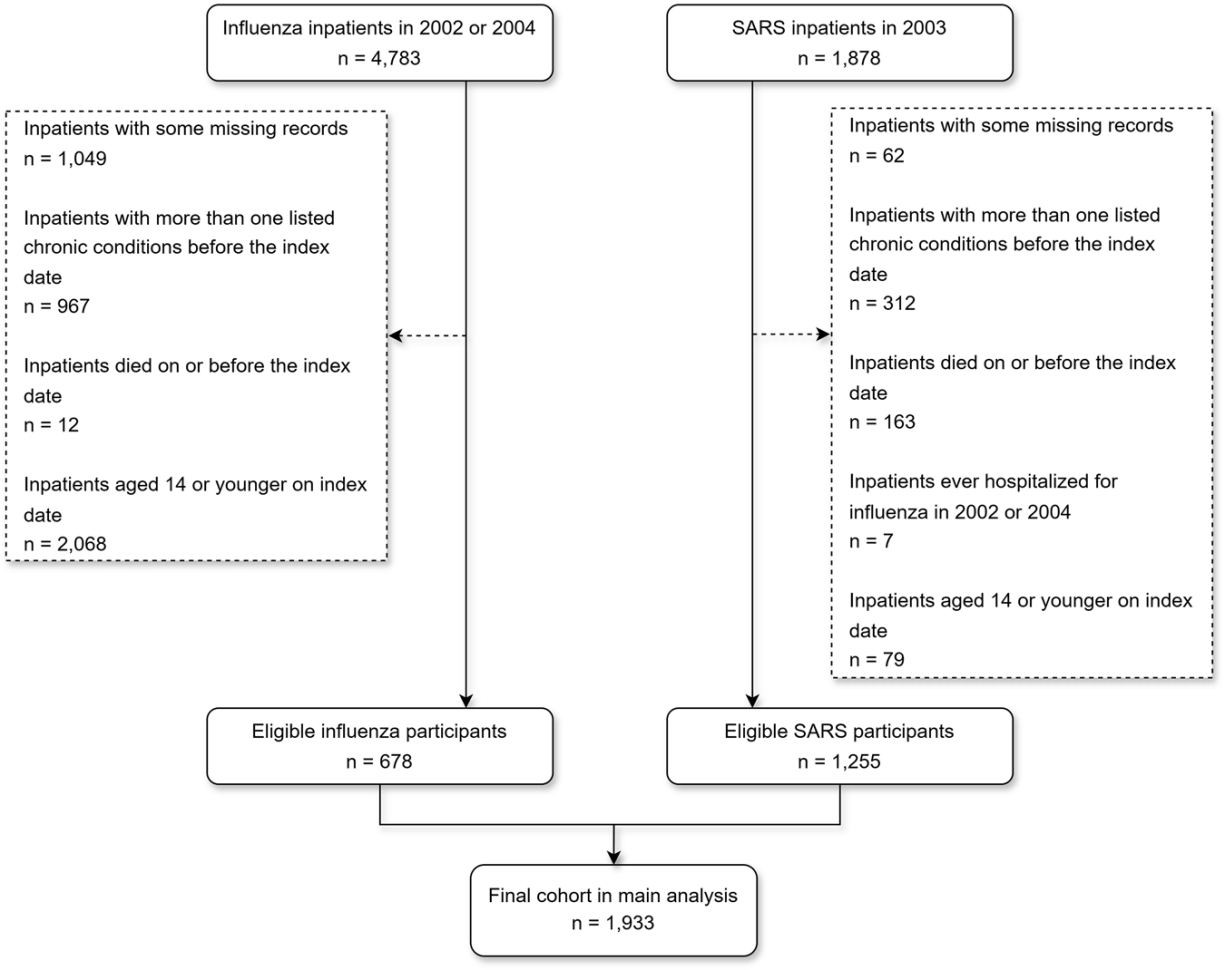
Cohort selection flowchart. This flowchart shows the process of retrospective cohort selection from the Hospital Authority’s database.

Figure 1 shows the procedures of cohort selection. After excluding patients who met exclusion criteria such as death on or before the index date or living with more than one chronic disease at the baseline, we eventually identified 678 influenza inpatients from 2002 or 2004 and 1255 SARS inpatients from 2003 to be included in the final study cohort.

### Cohort characteristics

Patient demographics and the history of chronic conditions and medications were summarized in Table 1. There were 340 (49.5%) males admitted with influenza and 496 (39.5%) males admitted with SARS. The mean age of influenza arm was 58.7 (Standard Deviation [SD] 24.40) and that of SARS group was 38.8 (SD 14.61). Five hundred and twenty-six patients had one chronic condition at baseline. The most common chronic conditions in the influenza group were chronic pulmonary disease (9.9%), hypertension (7.6%), diabetes (4.5%) and asthma (4.5%), while the chronic conditions with a higher proportion within SARS group were chronic pain (2.2%) and hypertension (2.1%). As for medications within one year before index date, most patients in both groups had a history of taking antibacterial drugs (Influenza group:87.3%, SARS group:99.2%) and antiviral drugs (Influenza group:51.2%, SARS group:93.6%). After IPTW, some characteristics like age, sex, and stroke at baseline between the 2 groups were still unbalanced, as indicated by an SMD or proportion difference of >0.1.

**Table 1:** Baseline cohort characteristics.

	Influenza	SARS	Influenza	SARS	
	Unweighted		Weighted		SMD
n	687	1255	1847	3129	–
Age [mean (SD)]	58.67 (24.40)	38.76 (14.61)	43.37 (24.44)	58.23 (22.20)	0.637
Sex: male (%)	340 (49.5)	496 (39.5)	862.1 (46.7)	1021.2 (32.6)	0.141
Chronic conditions (%)					
Chronic pulmonary disease	68 (9.9)	14 (1.1)	83.5 (4.5)	137.2 (4.4)	0.001
Hypertension	52 (7.6)	26 (2.1)	81.0 (4.4)	160.0 (5.1)	0.007
Diabetes	31 (4.5)	24 (1.9)	54.6 (3.0)	86.6 (2.8)	0.002
Chronic pain	27 (3.9)	27 (2.2)	34.8 (1.9)	71.0 (2.3)	0.004
Asthma	31 (4.5)	5 (0.4)	36.0 (1.9)	16.0 (0.5)	0.014
Stroke	21 (3.1)	6 (0.5)	26.8 (1.5)	623.7 (19.9)	0.184
Epilepsy	20 (2.9)	5 (0.4)	24.8 (1.3)	14.2 (0.5)	0.008
Depression	5 (0.7)	17 (1.4)	11.6 (0.6)	19.2 (0.6)	<0.001
Schizophrenia	14 (2.0)	3 (0.2)	17.7 (1.0)	6.5 (0.2)	0.008
Chronic heart failure	12 (1.7)	4 (0.3)	19.3 (1.0)	94.1 (3.0)	0.020
Chronic kidney disease	8 (1.2)	7 (0.6)	12.4 (0.7)	15.8 (0.5)	0.002
Atrial fibrillation	12 (1.7)	2 (0.2)	14.8 (0.8)	51.6 (1.6)	0.008
Cancer, non-metastatic	8 (1.2)	6 (0.5)	11.8 (0.6)	10.2 (0.3)	0.003
Peptic ulcer disease	10 (1.5)	4 (0.3)	14.4 (0.8)	13.5 (0.4)	0.004
Severe constipation	5 (0.7)	6 (0.5)	10.3 (0.6)	46.5 (1.5)	0.009
Dementia	6 (0.9)	3 (0.2)	9.2 (0.5)	21.1 (0.7)	0.002
Rheumatoid arthritis	3 (0.4)	4 (0.3)	102.4 (5.5)	33.3 (1.1)	0.044
Myocardial infraction	3 (0.4)	3 (0.2)	6.4 (0.3)	5.7 (0.2)	0.001
Cancer, lymphoma	5 (0.7)	0 (0.0)	5.0 (0.3)	0.0 (0.0)	0.003
Hypothyroidism	4 (0.6)	1 (0.1)	4.4 (0.2)	1.6 (0.1)	0.001
Parkinson	4 (0.6)	1 (0.1)	7.9 (0.4)	146.7 (4.7)	0.043
Alcohol misuse	1 (0.1)	2 (0.2)	2.6 (0.1)	3.3 (0.1)	<0.001
Cirrhosis	0 (0.0)	3 (0.2)	0.0 (0.0)	3.0 (0.1)	0.001
Cancer, metastatic	0 (0.0)	1 (0.1)	0.0 (0.0)	1.0 (0.0)	<0.001
Inflammatory bowel disease	0 (0.0)	1 (0.1)	0.0 (0.0)	1.0 (0.0)	<0.001
Psoriasis	1 (0.1)	0 (0.0)	1.0 (0.1)	0.0 (0.0)	0.001
Medications at baseline (%)					
Antibacterial drugs	600 (87.3)	1245 (99.2)	1748.6 (94.7)	2495.5 (79.8)	0.149
Antiviral drugs	352 (51.2)	1175 (93.6)	1399.7 (75.8)	1447.7 (46.3)	0.295
Calcium channel blockers	108 (15.7)	65 (5.2)	158.0 (8.6)	288.5 (9.2)	0.006
Diuretics	72 (10.5)	65 (5.2)	119.7 (6.5)	206.7 (6.6)	0.001
Beta blockers	72 (10.5)	56 (4.5)	113.3 (6.1)	320.3 (10.2)	0.041
Antiplatelets	73 (10.6)	39 (3.1)	107.4 (5.8)	675.0 (21.6)	0.158
Antidiabetic drugs	51 (7.4)	52 (4.1)	87.3 (4.7)	129.1 (4.1)	0.006
Renin-angiotensin-system agents	51 (7.4)	37 (2.9)	106.2 (5.7)	110.5 (3.5)	0.022
Insulins	15 (2.2)	62 (4.9)	45.0 (2.4)	76.2 (2.4)	<0.001
Nitrates	44 (6.4)	24 (1.9)	64.3 (3.5)	48.7 (1.6)	0.019
Lipid lowering agents	29 (4.2)	27 (2.1)	46.8 (2.5)	52.2 (1.7)	0.008
Antidepressants	21 (3.1)	26 (2.1)	38.5 (2.1)	72.5 (2.3)	0.002
Immunosuppressants	8 (1.2)	6 (0.5)	19.1 (1.0)	32.9 (1.1)	0.001
Oral anticoagulants	4 (0.6)	10 (0.8)	6.3 (0.3)	11.9 (0.4)	0.001
Antiarrhythmic drugs	5 (0.7)	5 (0.4)	11.5 (0.6)	66.0 (2.1)	0.015

### Weighted analysis of multimorbidity incidence

**Fig. 2:**
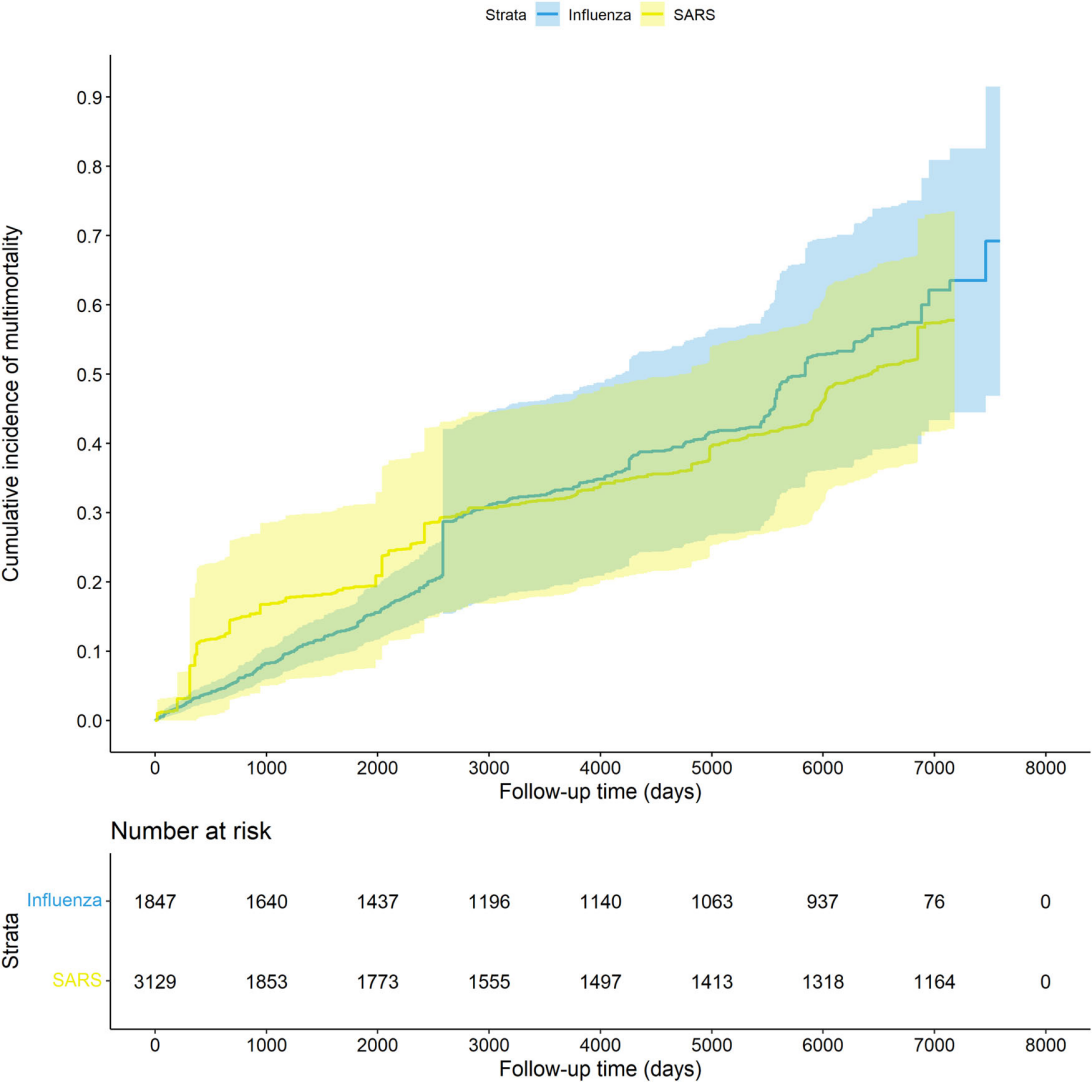
Cumulative multimorbidity incidence for SARS and influenza patients after inverseprobability of treatment weighting. This figure shows the cumulative incidence of multimorbidity over time for SARS (yellow plot) and influenza (blue plot) patients, with inverse probability of treatment weighting. The number at risk is indicated below the plot.

**Fig. 3:**
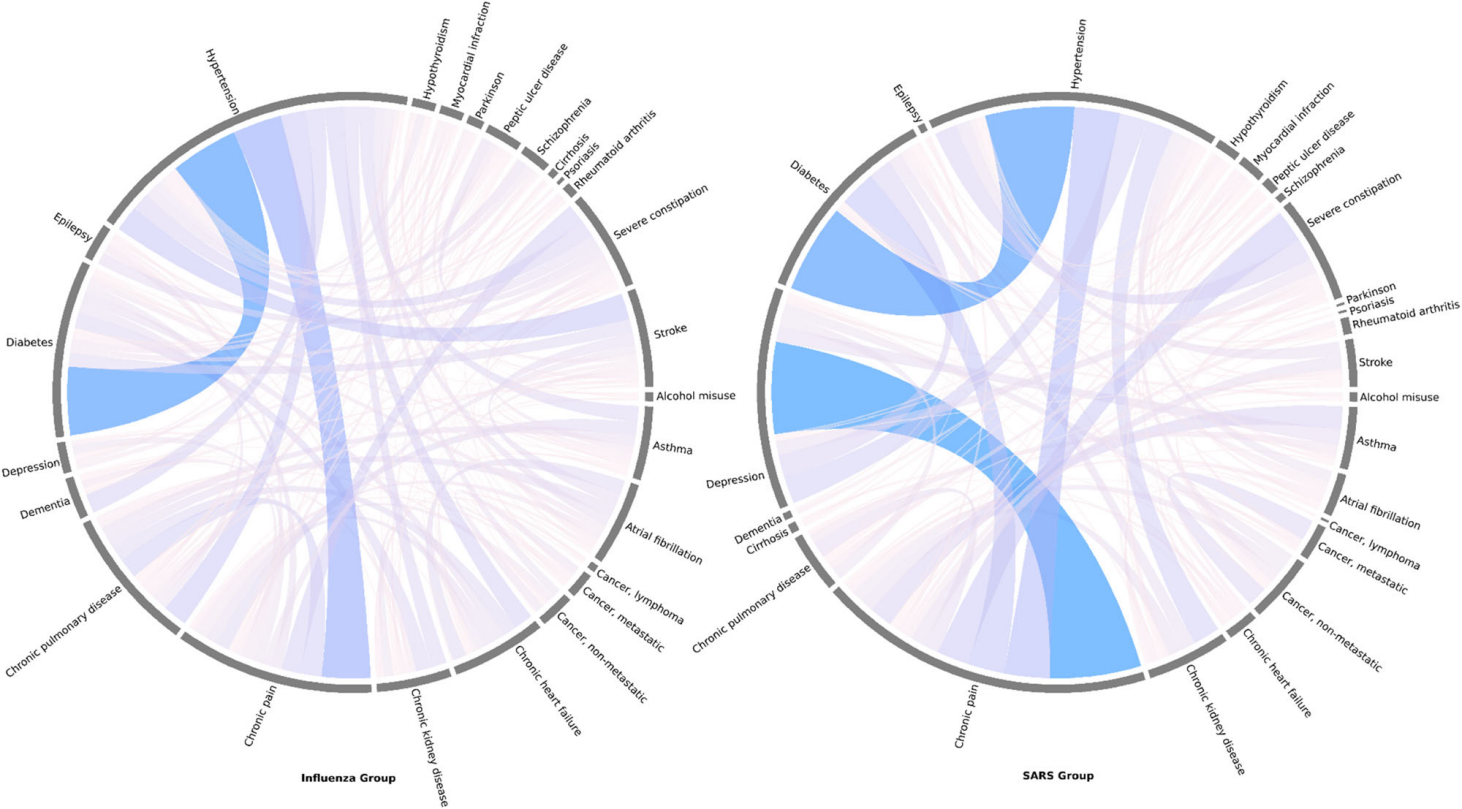
Disease combination patterns in influenza and SARS patients. This figure shows chord diagrams illustrating the patterns of disease combinations in influenza (left) and SARS (right) patients with multimorbidity.

A total of 376 influenza inpatients (54.7%), compared to 311 SARS inpatients (24.8%) developed multimorbidity during follow-up. The crude multimorbidity incidence rate per 100 person-years was 5.6 in influenza arm and 1.5 in SARS arm. As shown in Fig. 2, cumulative incidence of multimorbidity among SARS patients increased faster than that of influenza group in approximately the first 2700 days after discharge from hospital while in the subsequent years, the cumulative incidence of multimorbidity in the SARS arm became slightly lower than the influenza arm. Figure 3 showed chord diagrams by influenza and SARS groups exemplifying the relative frequencies (represented by ribbon area) of chronic condition pairings with a deeper color representing a higher frequency. Hypertension-diabetes was the most common chronic condition cooccurrence in both groups. However, the coexistence of depression and chronic pain was much more obvious among SARS inpatients.

As shown in Table 2, after IPTW and multivariable adjustment, patients of the SARS arm was shown to have a significantly lower multimorbidity incidence rate than those of influenza arm (IRR 0.78, 95%CI 0.70–0.86, *P* < 0.0001). Adjusted subgroup analysis showed no evidence of differences between women between the two arms (IRR 1.00, 95%CI 0.88–1.14, *P* = 0.9661), while the result for men were similar to the overall result (IRR 0.68, 95%CI 0.57–0.80, *P* < 0.0001). In age group-stratified analyses, those aged younger than 40 in the SARS arm had a significantly lower risk of multimorbidity than those in the influenza arm (IRR 0.86, 95%CI 0.77–0.96, *P* = 0.0091). No significant difference was estimated between the arms in those aged 40 years or older (IRR 0.97, 95%CI 0.76–1.23, *P* = 0.7865).

**Table 2:** Poisson regression comparing multimorbidity incidence between SARS-CoV-1 infection and influenza patients.

Group	No. of persons	No. of multimorbidity cases/No. person-years	No. of events per 100-person-years	Unweighted IRR (95% CI), *P*-value ^a b^	Weighted IRR (95% CI), *P*-value ^b^
Overall					
Influenza	687	376/6,760.91	5.6	Ref	Ref
SARS	1255	311/21,029.82	1.5	0.27 (0.23, 0.31), <0.0001	0.78 (0.70, 0.86), <0.0001
Women					
Influenza	347	205/3,492.60	5.9	Ref	Ref
SARS	759	201/12,845.80	1.6	0.27 (0.22, 0.32), <0.0001	1.00 (0.88, 1.14), 0.9661
Men					
Influenza	340	171/3,268.31	5.2	Ref	Ref
SARS	496	110/8,184.02	1.3	0.26 (0.20, 0.33), <0.0001	0.68 (0.57, 0.80), <0.0001
Age ≥ 40					
Influenza	496	333/3,571.28	9.3	Ref	Ref
SARS	535	227/7,519.67	3.0	0.32 (0.27, 0.38), <0.0001	0.86 (0.77, 0.96), 0.0091
Age < 40					
Influenza	191	43/3,189.63	1.3	Ref	Ref
SARS	730	84/13,510.14	0.6	0.46 (0.32, 0.67), <0.0001	0.97 (0.76, 1.23), 0.7865

^a^ IRR = incidence rate ratio; CI = confidence interval.

^b^ Propensity score-based inverse probability of treatment weighting was used to weight the sample according to age at index date, sex, medications, and baseline chronic disease. Covariates that were unbalanced after weighting were included in the multivariable adjustment in the regression model.

The results of the analyses of the secondary outcomes are shown in S3 Table. For specific diseases, analysis showed there is a significantly higher incidence of depression, diabetes, non-metastatic cancer, and chronic pain among hospitalized patients with SARS.

### Sensitivity analysis

In the sensitivity analysis using influenza patients hospitalized in 2003, despite fewer individuals compared to the primary analysis with 2002 and 2004 data, the incidence rate of multimorbidity remained consistent with the primary findings, showing no significant difference between the SARS and influenza groups (S4 Table). Moving the index date to one year after discharge, we found that the risk of multimorbidity was slightly lower in the SARS arm, similar with the results of main and subgroup analysis (S5 Table). In the sensitivity analysis regarding the cooccurrence of three chronic diseases as multimorbidity, results were similar to the main analyses for primary outcome except for the subgroup younger than 40 years (S6 Table). The different result, however, was based on only one outcome event in each of the two arms. No significant differences in multimorbidity incidence rates were observed by using full multivariable Poisson regression instead of IPTW to address the covariates (S7 Table). No notable deviations from the main findings are observed in the sensitivity analyses using a competing risk regression to adjust competing risks from mortality (S8 Table) and considering only antivirals and antibiotics during the current episode as the covariates (S9 Table). As shown in S10 Table, no significant impact from the seasonality of influenza on the main findings was observed (IRR 0.76, 95%CI 0.55–1.04, *P* = 0.0838). S11 Table shows the stratified results for different observation periods and supports the pattern shown in Fig. 2 that cumulative incidence of multimorbidity among SARS patients increased faster in earlier periods.

## Discussion

In this retrospective cohort study of individuals over two decades, we did not identify a higher multimorbidity incidence following a SARS-related hospitalization compared with an influenza-related hospitalization. We showed that multimorbidity incidence after SARS-related hospitalizations increased at a faster pace in the first couple of years, but the difference gradually became negligible over the long follow-up period. Sub-analyses by age and sex were largely consistent with the main findings, with sensitivity analyses using influenza cases from a different period, delaying the index date by one year, using three diseases as the threshold to define multimorbidity, adjusting for competing risks from all-cause mortality, and using an alternative covariates selection and adjustment approach all supporting the robustness of the main results. Nevertheless, we identified notable specific differences in the multimorbidity patterns between the two groups.

To the best of our knowledge, this is the first study examining the 20-year multimorbidity incidence of SARS survivors in comparison with influenza acquired in similar periods. It adds to other studies reporting on the clinical sequala of SARS^23,24^ and showed that, over the long term, overall multimorbidity was not higher in SARS survivors than influenza patients. It provides useful information on how acute viral infections with coronavirus may translate into chronic healthcare burden and it also represents a rare scientific inquiry to examine multimorbidity as an outcome which encompasses various diseases and disorders over a prolonged period.

The faster increase in multimorbidity incidence in the first few years of discharge from a SARS episode was likely due to health check-ups or follow-up consultations after the infection because of increased health awareness^25^. For instance, the higher incidence of diabetes, which typically requires health checks to diagnose, was one of the diseases the incidence rate of which was found to be higher in the SARS arm than in the influenza arm. Indeed, our study identified notable specific differences in the patterns of multimorbidity developing in SARS survivors compared to influenza patients. Specifically, SARS survivors were more prone to conditions like chronic pain, depression, and diabetes, while influenza patients showed greater risks of cardiovascular issues, such as atrial fibrillation and heart failure, as well as neurological conditions like dementia. These differences might reflect SARS’s intense inflammatory effects, potentially driving pain and mental health challenges, whereas influenza may worsen pre-existing cardiovascular and neurological vulnerabilities^15,26^. Consistent with previous research^27^, we found that the pattern of chronic pain – depression was more common among those discharged from a SARS episode, suggesting that types of care required over the long run after a respiratory infection may also differ across various sociodemographic and disease groups. These findings have important implications for the long-term management of patients with severe respiratory infections and suggest the need for tailored interventions to address the unique needs of different patient populations. The identification of specific multimorbidity patterns may also guide the development of targeted screening and prevention strategies for certain subgroups of patients^28^, which could ultimately lead to improved health outcomes and quality of life.

The strengths of this study include the use of a large population-based dataset with a long follow-up period, which allowed us to investigate the long-term consequences of respiratory infections. Additionally, we used a comprehensive definition of multimorbidity that considered a wide range of chronic diseases, which is important as the management of multimorbidity requires a holistic approach. However, our study also has some limitations. First, we were unable to account for unobserved potential confounders such as smoking and other lifestyle factors, which may have affected our results. Second, our study only included individuals who were hospitalized for respiratory infections, and therefore our findings may not be generalizable to patients who were not hospitalized or who were hospitalized for other reasons. Third, we were unable to distinguish between different types of influenza viruses, and therefore our comparison group may not have been entirely homogeneous. Fourth, potential underdiagnosis of chronic diseases due to variations in healthcare utilization patterns may underestimate their prevalence in both groups. However, these variations and potential underestimation are likely similar between them, with limited impact on the validity in our comparative analysis. Last, our study only included individuals Hong Kong where a predominantly ethnic Chinese population resides, and therefore our findings may not be generalizable to other populations or healthcare systems.

In conclusion, our study found that there was no higher long-term incidence of multimorbidity among SARS survivors compared with influenza patients. However, we identified notable differences in the patterns of multimorbidity developed in these two groups, which could be attributed to differences in baseline patient characteristics. These findings highlight the need for tailored interventions and targeted screening strategies to address the unique needs of different patient populations, which could ultimately lead to improved health outcomes and quality of life.

## Supplementary information

Supplementary Information

## Data Availability

Data is not available as the data custodian has not given permission for data sharing.
