# Supplementary material for: Multimorbidity incidence following hospitalization for SARS-CoV-1 infection or influenza over two decades: a territory-wide retrospective cohort study
Source: NPJ Prim Care Respir Med. 2025 Mar 25;35:18. doi: 10.1038/s41533-025-00424-y (PMC11937326; doi:10.1038/s41533-025-00424-y)
Supplement: Supplementary file 1 — Supplementary Information [file 41533_2025_424_MOESM1_ESM.docx]

## SUPPLEMENTAL TABLES

| **S1 Table.** List of chronic conditions for the definition of multimorbidity and corresponding diagnostic codes based on the International Classification of Diseases, Ninth Revision, Clinical Modification. | | |
| --- | --- | --- |
| Chronic conditions | International Classification of Diseases, Ninth Revision | International Classification of Primary Care |
| Alcohol misuse | 265.2, 291.1–291.3, 291.5–291.9, 303.0, 303.9, 305.0, 357.5, 425.5, 535.3, 571.0–571.3, 980, V11.3 | P15 |
| Asthma | 493 | R96 |
| Atrial fibrillation | 427.3 | K78 |
| Cancer, lymphoma | 200–202, 203.0, 238.6 | B72 |
| Cancer, metastatic | 196–199 | B74, D74, D76, D77, L71, N74, S77, T71, U75, U76, U77, W72, X77, Y78 |
| Cancer, non-metastatic (breast, cervical, colorectal, lung, prostate) | 153-154, 162-163, 174, 180, 185, 230.3-230.6, 231.2, 233.0-233.1, 233.4 | D75, R84, X75, X76, Y77 |
| Chronic heart failure | 398.91, 402.01, 402.11, 402.91, 404.01, 404.03, 404.11, 404.13, 404.91, 404.93, 425.4–425.9, 428 | K77 |
| Chronic kidney disease | 583, 584, 585, 586, 592, 593.9 | U14 |
| Chronic pain | 307.80, 307.89, 338.0, 338.2, 338.4, 719.41, 719.45 - 719.47, 719.49, 720.0, 720.2, 720.9, 721.0 - 721.4, 721.6, 721.8, 721.9, 722, 723.0, 723.1, 723.3 - 723.9, 724.0 - 724.6, 724.70, 724.79, 724.8, 724.9, 729.0 - 729.2, 729.4, 729.5 | A01 |
| Chronic pulmonary disease | 416.8, 416.9, 490–492, 494-505, 506.4, 508.1, 508.8 | R95 |
| Chronic viral hepatitis B | 70.2-70.3 | D72 |
| Cirrhosis | 571.2, 571.5, 571.6, 456.0, 456.1, 456.20, 456.21, 567.0, 567.2, 567.21, 567.29, 567.8, 567.9, 572.2, 572.4, 789.5 (Exclude 567.81, 567.82, 789.51) | - |
| Dementia | 290, 294.1, 331.2 | P70 |
| Depression | 296.2, 296.3, 296.5, 300.4, 309, 311 | P76 |
| Diabetes | 250 | T89-T90 |
| Epilepsy | 345 | N88 |
| Hypertension | 401-405 | K86-K87 |
| Hypothyroidism | 240.9, 243, 244, 246.1, 246.8 | T86 |
| Inflammatory bowel disease | 555, 556 | - |
| Irritable bowel syndrome | 564.1 (Exclude 153-154, 157, 183.0, 197.5, 198.6, 235.2, 239.0, 555-556, 571.2, 571.5, 577.1, 579) | D93 |
| Multiple sclerosis | 323, 340, 341.0, 341.9, 377.3 | N86 |
| Myocardial infarction | 410 | K75 |
| Parkinson’s disease | 332 | N87 |
| Peptic ulcer disease | 531.7, 531.9, 532.7, 532.9, 533.7, 533.9, 534.7, 534.9 | D86 |
| Peripheral vascular disease | 440.2 | K92 |
| Psoriasis | 696.1 | S91 |
| Rheumatoid arthritis | 446.5, 710.0–710.4, 714.0–714.2, 714.8, 725 | L88 |
| Schizophrenia | 295 | P72 |
| Severe constipation | 560.1, 560.30, 560.39, 560.9, 564.0, 569.83, 569.89 (Exclude 152-154, 158, 179-189, 197.5-197.6, 235.2, 239.0, 555-556, 568.0, 614.6, (560.9 if 789.01, 789.02, 789.06)) | D12 |
| Stroke or transient ischemic attack | 362.3, 430, 431, 433.x1, 434.x1, 435, 436 | K90 |

| **S2 Table.** List of types of medications at baseline and the corresponding British National Formulary codes. | |
| --- | --- |
| Medications | British National Formulary code |
| Renin-angiotensin-system agents | 2.5.5 |
| Beta blockers | 2.4 |
| Calcium channel blockers | 2.6.2 |
| Diuretics | 2.2 |
| Nitrates | 2.6.1 |
| Lipid lowering agents | 2.12 |
| Insulins | 6.1.1 |
| Antidiabetic drugs | 6.1.2 |
| Antiarrhythmic drugs | 2.3.2 |
| Oral anticoagulants | 2.8.2 |
| Antiplatelets | 2.9 |
| Steroid | 6.3.2 |
| Antidepressants | 4.3 |
| Antiviral drugs | 5.3 |
| Antibacterial drugs | 5.1 |
| Immunosuppressants | 8.2 |

| **S3 Table.** Analysis for specific disease outcomes | | | | | |
| --- | --- | --- | --- | --- | --- |
| Chronic condition | No. of persons | No. of multimorbidity cases/No. person-years | No. of events per 1,000-person-years | Unweighted IRR (95% CI), P-value ^a b^ | Weighted IRR (95% CI), P-value ^b^ |
| Alcohol misuse |  |  |  |  |  |
| Influenza | 686 | 4/6,754.25 | 0.6 | Ref | Ref |
| SARS | 1253 | 5/21,020.16 | 0.2 | 0.65 (0.18, 2.43), 0.5241 | 1.63 (0.42, 6.41), 0.4816 |
| Asthma |  |  |  |  |  |
| Influenza | 656 | 14/6,469.86 | 2.2 | Ref | Ref |
| SARS | 1251 | 22/20,979.61 | 1.0 | 0.78 (0.40, 1.53), 0.4747 | 1.42 (0.80, 2.52), 0.2248 |
| Atrial fibrillation |  |  |  |  |  |
| Influenza | 675 | 30/6,687.85 | 4.5 | Ref | Ref |
| SARS | 1253 | 14/21,012.85 | 0.7 | 0.23 (0.12, 0.44), <0.0001 | 0.30 (0.22, 0.40), <0.0001 |
| Cancer, lymphoma |  |  |  |  |  |
| Influenza | 682 | 1/6,727.72 | 0.1 | Ref | Ref |
| SARS | 1255 | 1/21,029.82 | 0 | 0.52 (0.03, 8.29), 0.6421 | 1.11 (0.10, 11.78), 0.9338 |
| Cancer, metastatic |  |  |  |  |  |
| Influenza | 687 | 13/6,760.91 | 1.9 | Ref | Ref |
| SARS | 1254 | 15/21,029.8 | 0.7 | 0.60 (0.29, 1.26), 0.1779 | 0.60 (0.35, 1.02), 0.0596 |
| Cancer, non-metastatic |  |  |  |  |  |
| Influenza | 679 | 16/6,710.98 | 2.4 | Ref | Ref |
| SARS | 1249 | 33/20,968.93 | 1.6 | 1.07 (0.59, 1.94), 0.8308 | 1.63 (1.01, 2.64), 0.0452 |
| Chronic heart failure |  |  |  |  |  |
| Influenza | 675 | 37/6,710.68 | 5.5 | Ref | Ref |
| SARS | 1251 | 7/21,019.87 | 0.3 | 0.09 (0.04, 0.21), <0.0001 | 0.31 (0.18, 0.56), <0.0001 |
| Chronic kidney disease |  |  |  |  |  |
| Influenza | 679 | 38/6,744.63 | 5.6 | Ref | Ref |
| SARS | 1248 | 31/20,936.5 | 1.5 | 0.41 (0.26, 0.66), 0.0003 | 0.63 (0.44, 0.89), 0.0096 |
| Chronic pain |  |  |  |  |  |
| Influenza | 660 | 95/6,607.52 | 14.4 | Ref | Ref |
| SARS | 1228 | 203/20,724.66 | 9.8 | 1.11 (0.87, 1.42), 0.3886 | 1.52 (1.29, 1.79), <0.0001 |
| Chronic pulmonary disease |  |  |  |  |  |
| Influenza | 619 | 34/6,480.19 | 5.2 | Ref | Ref |
| SARS | 1241 | 23/20,968.32 | 1.1 | 0.31 (0.18, 0.53), <0.0001 | 0.33 (0.22, 0.49), <0.0001 |
| Cirrhosis |  |  |  |  |  |
| Influenza | 687 | 6/6,760.91 | 0.9 | Ref | Ref |
| SARS | 1252 | 2/20,997.68 | 0.1 | 0.17 (0.04, 0.86), 0.0321 | 0.20 (0.06, 0.73), 0.0146 |
| Dementia |  |  |  |  |  |
| Influenza | 681 | 19/6,748.16 | 2.8 | Ref | Ref |
| SARS | 1252 | 3/21,017.84 | 0.1 | 0.08 (0.02, 0.27), <0.0001 | 0.19 (0.07, 0.53), 0.0013 |
| Depression |  |  |  |  |  |
| Influenza | 682 | 19/6,713.60 | 2.8 | Ref | Ref |
| SARS | 1238 | 128/20,799.36 | 6.2 | 3.77 (2.33, 6.11), <0.0001 | 1.83 (1.39, 2.40), <0.0001 |
| Diabetes |  |  |  |  |  |
| Influenza | 656 | 48/6,584.94 | 7.3 | Ref | Ref |
| SARS | 1231 | 71/20,768.26 | 3.4 | 0.74 (0.52, 1.07), 0.1150 | 1.51 (1.19, 1.92), 0.0008 |
| Epilepsy |  |  |  |  |  |
| Influenza | 667 | 8/6,543.46 | 1.2 | Ref | Ref |
| SARS | 1250 | 2/20,945.62 | 0.1 | 0.13 (0.03, 0.60), 0.0089 | 0.24 (0.13, 0.45), <0.0001 |
| Hypertension |  |  |  |  |  |
| Influenza | 635 | 130/6,471.89 | 20.1 | Ref | Ref |
| SARS | 1229 | 141/20,793.18 | 6.8 | 0.49 (0.39, 0.62), <0.0001 | 0.94 (0.79, 1.12), 0.5073 |
| Hypothyroidism |  |  |  |  |  |
| Influenza | 683 | 7/6,751.60 | 1.0 | Ref | Ref |
| SARS | 1254 | 12/21,010.38 | 0.6 | 0.89 (0.35, 2.26), 0.8050 | 0.12 (0.07, 0.20), <0.0001 |
| Myocardial infraction |  |  |  |  |  |
| Influenza | 684 | 9/6,746.45 | 1.3 | Ref | Ref |
| SARS | 1252 | 9/21,024.97 | 0.4 | 0.52 (0.21, 1.31), 0.1646 | 1.74 (0.91, 3.31), 0.0941 |
| Parkinson |  |  |  |  |  |
| Influenza | 683 | 7/6,740.68 | 1.0 | Ref | Ref |
| SARS | 1254 | 1/21,028.58 | 0 | 0.07 (0.01, 0.60), 0.0147 | 0.11 (0.02, 0.68), 0.0172 |
| Peptic ulcer disease |  |  |  |  |  |
| Influenza | 677 | 8/6,713.39 | 1.2 | Ref | Ref |
| SARS | 1251 | 4/20,982.7 | 0.2 | 0.26 (0.08, 0.85), 0.0265 | 0.90 (0.44, 1.84), 0.7755 |
| Psoriasis |  |  |  |  |  |
| Influenza | 686 | 1/6,743.06 | 0.1 | Ref | Ref |
| SARS | 1255 | 1/21,029.82 | 0 | 0.52 (0.03, 8.34), 0.6455 | 1.09 (0.08, 14.49), 0.9497 |
| Rheumatoid arthritis |  |  |  |  |  |
| Influenza | 684 | 3/6,746.86 | 0.4 | Ref | Ref |
| SARS | 1251 | 5/20,987.47 | 0.2 | 0.87 (0.21, 3.64), 0.8486 | 1.05 (0.30, 3.66), 0.9429 |
| Schizophrenia |  |  |  |  |  |
| Influenza | 673 | 4/6,613.16 | 0.6 | Ref | Ref |
| SARS | 1251 | 4/20,995.14 | 0.2 | 0.51 (0.13, 2.05), 0.3443 | 0.12 (0.05, 0.33), <0.0001 |
| Severe constipation |  |  |  |  |  |
| Influenza | 682 | 50/6,701.70 | 7.5 | Ref | Ref |
| SARS | 1249 | 49/20,987.17 | 2.3 | 0.50 (0.34, 0.74), 0.0006 | 0.62 (0.47, 0.81), 0.0005 |
| Stroke |  |  |  |  |  |
| Influenza | 666 | 38/6,666.72 | 5.7 | Ref | Ref |
| SARS | 1249 | 24/21,006.66 | 1.1 | 0.31 (0.19, 0.52), <0.0001 | 0.46 (0.29, 0.71), 0.0005 |
| ^a^ IRR = incidence rate ratio; CI = confidence interval | | | | | |
| ^b^ Propensity score-based inverse probability of treatment weighting was used to weight the sample according to age at index date, sex, medications, and baseline chronic disease | | | | | |

| **S4 Table.** Sensitivity analysis using influenza patients hospitalized in 2003 instead of 2002 and 2004. | | | | | |
| --- | --- | --- | --- | --- | --- |
| Group | No. of persons | No. of multimorbidity cases/No. person-years | No. of events per 100-person-years | Unweighted IRR (95% CI), P-value ^a b^ | Weighted IRR (95% CI), P-value ^b^ |
| Overall |  |  |  |  |  |
| Influenza | 193 | 79/2,406.59 | 3.3 | Ref | Ref |
| SARS | 1255 | 311/21,029.82 | 1.5 | 0.45 (0.35, 0.58), <0.0001 | 0.92 (0.80, 1.06), 0.2572 |
| Women |  |  |  |  |  |
| Influenza | 93 | 41/1,102.31 | 3.7 | Ref | Ref |
| SARS | 759 | 201/12,845.80 | 1.6 | 0.42 (0.30, 0.59), <0.0001 | 0.80 (0.67, 0.95), 0.0117 |
| Men |  |  |  |  |  |
| Influenza | 100 | 38/1,304.28 | 2.9 | Ref | Ref |
| SARS | 496 | 110/8,184.02 | 1.3 | 0.46 (0.32, 0.67), <0.0001 | 1.19 (0.94, 1.51), 0.1477 |
| Age ≥ 40 |  |  |  |  |  |
| Influenza | 119 | 13/1,112.86 | 1.2 | Ref | Ref |
| SARS | 525 | 84/7,519.67 | 1.1 | 0.51 (0.39, 0.67), <0.0001 | 0.90 (0.76, 1.06), 0.1998 |
| Age < 40 |  |  |  |  |  |
| Influenza | 74 | 66/1,293.73 | 5.1 | Ref | Ref |
| SARS | 730 | 227/13,510.14 | 1.7 | 0.62 (0.35, 1.11), 0.1073 | 0.84 (0.64, 1.10), 0.2096 |
| ^a^ IRR = incidence rate ratio; CI = confidence interval | | | | | |
| ^b^ Propensity score-based inverse probability of treatment weighting was used to weight the sample according to age at index date, sex, medications, and baseline chronic disease | | | | | |

| **S5 Table.** Sensitivity analysis using one year after discharge as the index date for observation. | | | | | |
| --- | --- | --- | --- | --- | --- |
| Group | No. of persons | No. of multimorbidity cases/No. person-years | No. of events per 100-person-years | Unweighted IRR (95% CI), P-value ^a b^ | Weighted IRR (95% CI), P-value ^b^ |
| Overall |  |  |  |  |  |
| Influenza | 671 | 355/6,669.27 | 5.3 | Ref | Ref |
| SARS | 1273 | 310/20,404.48 | 1.5 | 0.29 (0.25, 0.33), <0.0001 | 0.81 (0.72, 0.90), 0.0002 |
| Women |  |  |  |  |  |
| Influenza | 348 | 201/3,430.75 | 5.9 | Ref | Ref |
| SARS | 777 | 202/12,457.56 | 1.6 | 0.28 (0.23, 0.34), <0.0001 | 0.77 (0.67, 0.89), 0.0004 |
| Men |  |  |  |  |  |
| Influenza | 323 | 154/3,238.51 | 4.8 | Ref | Ref |
| SARS | 496 | 108/7,946.92 | 1.4 | 0.29 (0.22, 0.37), <0.0001 | 0.81 (0.68, 0.98), 0.0291 |
| Age ≥ 40 |  |  |  |  |  |
| Influenza | 460 | 308/3,319.43 | 9.3 | Ref | Ref |
| SARS | 557 | 230/7,793.77 | 3.0 | 0.32 (0.27, 0.38), <0.0001 | 0.86 (0.75, 0.97), 0.0149 |
| Age < 40 |  |  |  |  |  |
| Influenza | 211 | 47/3,349.84 | 1.4 | Ref | Ref |
| SARS | 716 | 80/12,610.72 | 0.6 | 0.45 (0.32, 0.65), <0.0001 | 0.69 (0.53, 0.89), 0.0040 |
| ^a^ IRR = incidence rate ratio; CI = confidence interval | | | | | |
| ^b^ Propensity score-based inverse probability of treatment weighting was used to weight the sample according to age at index date, sex, medications, and baseline chronic disease | | | | | |

| **S6 Table.** Sensitivity analysis using three diseases as the threshold for the definition of multimorbidity. | | | | | |
| --- | --- | --- | --- | --- | --- |
| Group | No. of persons | No. of multimorbidity cases/No. person-years | No. of events per 100-person-years | Unweighted IRR (95% CI), P-value ^a b^ | Weighted IRR (95% CI), P-value ^b^ |
| Overall |  |  |  |  |  |
| Influenza | 687 | 231/7,784.30 | 3.0 | Ref | Ref |
| SARS | 1259 | 163/22,436.85 | 0.7 | 0.24 (0.20, 0.30), <0.0001 | 0.66 (0.58, 0.75), <0.0001 |
| Women |  |  |  |  |  |
| Influenza | 347 | 134/4,020.95 | 3.3 | Ref | Ref |
| SARS | 759 | 102/13,690.47 | 0.7 | 0.22 (0.17, 0.29), <0.0001 | 0.89 (0.75, 1.05), 0.1706 |
| Men |  |  |  |  |  |
| Influenza | 340 | 97/3,763.35 | 2.6 | Ref | Ref |
| SARS | 500 | 61/8,746.37 | 0.7 | 0.27 (0.20, 0.37), <0.0001 | 0.69 (0.54, 0.87), 0.0023 |
| Age ≥ 40 |  |  |  |  |  |
| Influenza | 496 | 220/4,374.10 | 5.0 | Ref | Ref |
| SARS | 527 | 130/8,406.83 | 1.5 | 0.31 (0.25, 0.38), <0.0001 | 0.76 (0.66, 0.88), 0.0003 |
| Age < 40 |  |  |  |  |  |
| Influenza | 191 | 11/3,410.21 | 0.3 | Ref | Ref |
| SARS | 732 | 33/14,030.02 | 0.2 | 0.73 (0.37, 1.44), 0.3644 | 5.10 (2.89, 9.01), <0.0001 |
| ^a^ IRR = incidence rate ratio; CI = confidence interval | | | | | |
| ^b^ Propensity score-based inverse probability of treatment weighting was used to weight the sample according to age at index date, sex, medications, and baseline chronic disease | | | | | |

| **S7 Table.** Sensitivity analysis using full multivariable adjustment instead of inverse probability of treatment weighting to address covariates. | | | | | |
| --- | --- | --- | --- | --- | --- |
| Group | No. of persons | No. of multimorbidity cases/No. person-years | No. of events per 100-person-years | Crude IRR (95% CI), P-value ^a^ | Adjusted IRR (95% CI), P-value |
| Overall |  |  |  |  |  |
| Influenza | 687 | 376/6,760.91 | 5.6 | Ref | Ref |
| SARS | 1255 | 311/21,029.82 | 1.5 | 0.27 (0.23, 0.31), <0.0001 | 0.81 (0.65, 1.00), 0.0553 |
| Women |  |  |  |  |  |
| Influenza | 347 | 205/3,492.60 | 5.9 | Ref | Ref |
| SARS | 759 | 201/12,845.80 | 1.6 | 0.27 (0.22, 0.32), <0.0001 | 0.91 (0.68, 1.22), 0.5338 |
| Men |  |  |  |  |  |
| Influenza | 340 | 171/3,268.31 | 5.2 | Ref | Ref |
| SARS | 496 | 110/8,184.02 | 1.3 | 0.26 (0.20, 0.33), <0.0001 | 0.74 (0.52, 1.04), 0.0804 |
| Age ≥ 40 |  |  |  |  |  |
| Influenza | 496 | 333/3,571.28 | 9.3 | Ref | Ref |
| SARS | 535 | 227/7,519.67 | 3.0 | 0.32 (0.27, 0.38), <0.0001 | 0.74 (0.58, 0.95), 0.0158 |
| Age < 40 |  |  |  |  |  |
| Influenza | 191 | 43/3,189.63 | 1.3 | Ref | Ref |
| SARS | 730 | 84/13,510.14 | 0.6 | 0.46 (0.32, 0.67), <0.0001 | 0.73 (0.38, 1.39), 0.3396 |
| ^a^ IRR = incidence rate ratio; CI = confidence interval | | | | | |

| **S8 Table.** Sensitivity analysis using full multivariable competing risk regression to adjust for the competing risk from all-cause mortality | | | | | |
| --- | --- | --- | --- | --- | --- |
| Group | No. of persons | No. of multimorbidity cases/No. person-years | No. of events per 100-person-years | Crude HR (95% CI), P-value ^a^ | Adjusted HR (95% CI), P-value |
| Overall |  |  |  |  |  |
| Influenza | 687 | 376/6,760.91 | 5.6 | Ref | Ref |
| SARS | 1255 | 311/21,029.82 | 1.5 | 0.26 (0.23, 0.31), <0.001 | 0.73 (0.59, 0.91), 0.005 |
| Women |  |  |  |  |  |
| Influenza | 347 | 205/3,492.60 | 5.9 | Ref | Ref |
| SARS | 759 | 201/12,845.80 | 1.6 | 0.25 (0.21, 0.31), <0.001 | 0.81 (0.60, 1.09), 0.200 |
| Men |  |  |  |  |  |
| Influenza | 340 | 171/3,268.31 | 5.2 | Ref | Ref |
| SARS | 496 | 110/8,184.02 | 1.3 | 0.27 (0.21, 0.34), <0.001 | 0.68 (0.48, 0.96), 0.027 |
| Age ≥ 40 |  |  |  |  |  |
| Influenza | 496 | 333/3,571.28 | 9.3 | Ref | Ref |
| SARS | 535 | 227/7,519.67 | 3.0 | 0.30 (0.25, 0.36), <0.001 | 0.67 (0.53, 0.86), 0.002 |
| Age < 40 |  |  |  |  |  |
| Influenza | 191 | 43/3,189.63 | 1.3 | Ref | Ref |
| SARS | 730 | 84/13,510.14 | 0.6 | 0.45 (0.31, 0.65), <0.001 | 0.68 (0.47, 0.99), 0.046 |
| ^a^ HR = Hazard ratio; CI = confidence interval | | | | | |

| **S9 Table.** Sensitivity analysis: Poisson regression comparing multimorbidity incidence between SARS-CoV-1 infection and influenza patients considering antivirals and antibiotics only during the current episode | | | | | |
| --- | --- | --- | --- | --- | --- |
| Group | No. of persons | No. of multimorbidity cases/No. person-years | No. of events per 100-person-years | Unweighted IRR (95% CI), P-value ^a b^ | Weighted IRR (95% CI), P-value ^b^ |
| Overall |  |  |  |  |  |
| Influenza | 687 | 376/6,760.91 | 5.6 | Ref | Ref |
| SARS | 1255 | 311/21,029.82 | 1.5 | 0.27 (0.23, 0.31), <0.0001 | 0.78 (0.70, 0.87), <0.0001 |
| Women |  |  |  |  |  |
| Influenza | 347 | 205/3,492.60 | 5.9 | Ref | Ref |
| SARS | 759 | 201/12,845.80 | 1.6 | 0.27 (0.22, 0.32), <0.0001 | 0.85 (0.75, 0.97), 0.0131 |
| Men |  |  |  |  |  |
| Influenza | 340 | 171/3,268.31 | 5.2 | Ref | Ref |
| SARS | 496 | 110/8,184.02 | 1.3 | 0.26 (0.20, 0.33), <0.0001 | 0.66 (0.56, 0.79), <0.0001 |
| Age ≥ 40 |  |  |  |  |  |
| Influenza | 496 | 333/3,571.28 | 9.3 | Ref | Ref |
| SARS | 535 | 227/7,519.67 | 3.0 | 0.32 (0.27, 0.38), <0.0001 | 0.86 (0.77, 0.96), 0.0077 |
| Age < 40 |  |  |  |  |  |
| Influenza | 191 | 43/3,189.63 | 1.3 | Ref | Ref |
| SARS | 730 | 84/13,510.14 | 0.6 | 0.46 (0.32, 0.67), <0.0001 | 0.81 (0.63, 1.05), 0.1115 |
| ^a^ IRR = incidence rate ratio; CI = confidence interval | | | | | |
| ^b^ Propensity score-based inverse probability of treatment weighting was used to weight the sample according to age at index date, sex, medications, and baseline chronic disease. Covariates that were unbalanced after weighting were included in the multivariable adjustment in the regression model. | | | | | |

| **S10 Table.** Sensitivity analysis using full multivariable adjustment with seasonality influenza stratification | | | | | |
| --- | --- | --- | --- | --- | --- |
| Group | No. of persons | No. of multimorbidity cases/No. person-years | No. of events per 100-person-years | Crude IRR (95% CI), P-value ^a^ | Adjusted IRR (95% CI), P-value |
| Overall |  |  |  |  |  |
| Non-peak season influenza | 216 | 118/2212.20 | 5.3 | Ref | Ref |
| Peak season influenza | 471 | 258/4548.71 | 5.7 | 1.06 (0.86, 1.33), 0.5805 | 0.88 (0.70, 1.11), 0.2603 |
| SARS | 1255 | 311/21,029.82 | 1.5 | 0.28 (0.23, 0.34), <0.0001 | 0.76 (0.55, 1.04), 0.0838 |
| ^a^ IRR = incidence rate ratio; CI = confidence interval | | | | | |

| **S11 Table.** Sensitivity analysis: Poisson regression comparing multimorbidity incidence between SARS-CoV-1 infection and influenza patients by changing index date and follow-up period | | | | | |
| --- | --- | --- | --- | --- | --- |
| Follow-up period | No. of persons | No. of multimorbidity cases/No. person-years | No. of events per 100-person-years | Unweighted IRR (95% CI), P-value ^a b^ | Weighted IRR (95% CI), P-value ^b^ |
| <1 |  |  |  |  |  |
| Influenza | 687 | 43/653.61 | 6.6 | Ref | Ref |
| SARS | 1255 | 17/1,237.97 | 1.4 | 0.21 (0.12, 0.37), <0.0001 | 1.33 (0.97, 1.84), 0.0809 |
| 1-5 |  |  |  |  |  |
| Influenza | 651 | 136/2,196.28 | 6.2 | Ref | Ref |
| SARS | 1152 | 47/4,463.26 | 1.1 | 0.17 (0.12, 0.24), <0.0001 | 0.89 (0.70, 1.14), 0.3696 |
| 6-10 |  |  |  |  |  |
| Influenza | 419 | 77/1,451.72 | 5.3 | Ref | Ref |
| SARS | 1102 | 37/4,298.95 | 0.9 | 0.16 (0.11, 0.24), <0.0001 | 0.61 (0.45, 0.83), 0.0015 |
| 11-20 |  |  |  |  |  |
| Influenza | 308 | 101/2,191.22 | 4.6 | Ref | Ref |
| SARS | 1046 | 142/8,778.43 | 1.6 | 0.35 (0.27, 0.45), <0.0001 | 0.59 (0.49, 0.70), <0.0001 |
| ^a^ IRR = incidence rate ratio; CI = confidence interval | | | | | |
| ^b^ Propensity score-based inverse probability of treatment weighting was used to weight the sample according to age at index date, sex, medications, and baseline chronic disease. Covariates that were unbalanced after weighting were included in the multivariable adjustment in the regression model. | | | | | |
